# Extracellular vesicles as biomarkers for endometrial cancer – A systematic review

**DOI:** 10.1016/j.tranon.2025.102543

**Published:** 2025-09-22

**Authors:** Eva Baxter, Soumyalekshmi Nair, Zoe West, Carlos Salomon, Andreas Obermair

**Affiliations:** a Queensland Centre for Gynaecological Cancer Research, The University of Queensland, UQ Centre for Clinical Research, Brisbane QLD 4029, Australia; b Translational Extracellular Vesicles in Obstetrics and Gynae-Oncology Group, The University of Queensland, UQ Centre for Clinical Research, Brisbane QLD 4029, Australia; c UQ Centre for Extracellular Vesicle Nanomedicine, The University of Queensland, Brisbane QLD 4029, Australia

**Keywords:** Endometrial cancer, Extracellular vesicles, Biomarkers, Systematic review

## Abstract

**Background:**

The current method for diagnosing endometrial cancer and monitoring treatment response is invasive and fraught with both interobserver and intraobserver variability. Less invasive and more reproducible methods are desirable. This systematic review examines the evidence for extracellular vesicles as minimally invasive biomarkers for endometrial cancer.

**Methods:**

PubMed, Embase and Web of Science were searched for studies reporting extracellular vesicles as biomarkers in females with endometrial cancer. Risk of bias was assessed using the QUADAS-2 tool. A descriptive synthesis of biomarkers reported in the included studies was conducted.

**Results:**

Of the 680 unique records reviewed, 23 studies were included. All 23 studies investigated extracellular vesicles as diagnostic biomarkers. Ten extracellular vesicle-associated biomarkers were consistently reported to be differentially abundant between endometrial cancer cases and controls in multiple independent studies and hence may be putative diagnostic biomarkers. Levels of extracellular vesicles, LGALS3BP (galectin 3 binding protein), miR-15a-5p, and miR-21–3p were elevated, while levels of miR-26a-5p, miR-130a-3p, miR-139, miR-219a-5p, miR-222–3p, and miR-885 were decreased in endometrial cancer cases versus controls. Seven studies also investigated extracellular vesicles as prognostic biomarkers, but no biomarker was reported as prognostic in more than one study.

**Conclusion:**

Of the ten putative diagnostic biomarkers, extracellular vesicle-associated miR-21–3p, miR-26a-5p, miR-130a-3p, miR-139 and miR-219a-5p are the most promising as their expression in extracellular vesicle preparations appears to reflect that in endometrial tissue. However, there are significant concerns regarding study quality, limited adherence to consensus recommendations on extracellular vesicle research and lack of evidence supporting biomarkers being encapsulated within extracellular vesicles.

## Highlights

• Less invasive and more reproducible biomarkers are needed for endometrial cancer.
• Extracellular vesicles are promising minimally invasive biomarkers.
• The clinical potential of extracellular vesicles needs to be validated.

## Graphical abstract

**Figure:**
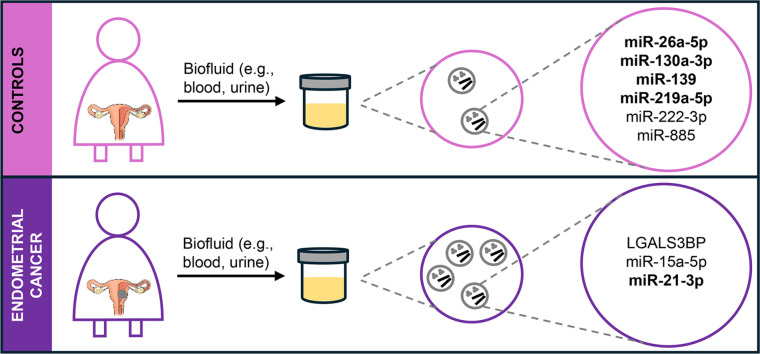

## Introduction

Endometrial cancer is the sixth most common cancer in females with 420,242 new cases and 97,704 deaths worldwide in 2022 [1]. Diagnosis of endometrial cancer and monitoring of treatment response require a tissue biopsy. However, tissue collection is an invasive procedure that can yield insufficient or low-quality tissue [2]. Furthermore, histopathological assessment of biopsies is challenging and subjective with significant interobserver and intraobserver variability, limiting its prognostic significance [3,4]. These limitations underscore the need for novel minimally invasive biomarkers that can reduce the burden of diagnostic tests and enhance diagnostic and prognostic precision and reproducibility.

Extracellular vesicles have shown promise as circulating biomarkers for several cancer types and are of particular interest due to their ability to detect cancer at an early stage and by minimally invasive methods [[5], [6], [7]]. ‘Extracellular vesicle’ is an umbrella term that encompasses particles encapsulated by a lipid bilayer that are released from cells [8,9]. These include exosomes that originate from the endosomal system and microvesicles and ectosomes that originate from the plasma membrane [8]. Due to the mixed origin of extracellular vesicle preparations and the lack of subtype-specific markers, the International Society for Extracellular Vesicles (ISEV) has recommended using size to categorize extracellular vesicles, designating them as small and large extracellular vesicles [9]. Extracellular vesicles can be isolated from biological fluids and contain a variety of bioactive molecules (e.g., proteins, micro RNAs (miRNAs)) that are protected from enzymatic degradation [10,11]. They also carry cellular markers from difficult-to-access anatomical sites, making them attractive candidates as minimally invasive biomarkers of disease [[12], [13], [14]]. The aim of this study is to systematically review the evidence for extracellular vesicles as minimally invasive biomarkers for endometrial cancer.

## Materials and methods

### Search strategy

PubMed, Embase and Web of Science were searched for studies reporting extracellular vesicles as biomarkers for endometrial cancer. Search terms were: (“extracellular vesicles”, “exosomes”, “ectosomes”, “EVs” or “microvesicles”) and (“endometrial”, “uterine” or “uterus”) and (“cancer”, “carcinoma”, “adenocarcinoma”, “neoplasia” or “hyperplasia”). This review was conducted in accordance with the Preferred Reporting Items for Systematic Reviews and Meta-Analyses guidelines [15]. The study protocol was registered in the International Prospective Register of Systematic Reviews (PROSPERO ID CRD42024521251).

### Study selection

All records published until 1 January 2024 were included. Duplicate records or records that were identified in more than one database were excluded. Next, records that were not in English or were not original research articles were excluded. The titles and abstracts were then screened against inclusion and exclusion criteria before full-text articles were assessed for eligibility. Any article reporting extracellular vesicles as biomarkers in females with endometrial cancer was included. Biomarkers could be of any type, including susceptibility/risk, diagnostic, monitoring, prognostic, predictive, response or safety [16]. Articles only reporting mechanistic, *in vitro* or *in silico* studies were excluded.

### Data extraction and synthesis

For each included study, data extracted included study design, study country, population, biofluid from which extracellular vesicles were isolated, extracellular vesicle isolation and characterization method, method for identifying biomarkers, significant biomarkers, biomarker type and details, including statistical information (p-value and performance such as AUC (area under the receiver operator characteristic curve)). A descriptive synthesis of extracellular vesicle biomarkers reported in the included studies was conducted.

### Risk of bias

Risk of bias was assessed using the QUADAS-2 tool for the following domains: patient selection, index test (extracellular vesicle biomarker), reference standard (histopathological diagnosis) and flow and timing [17]. All four domains were used to assess risk of bias, whereas only the first three domains were used to assess applicability concerns.

## Results

### Search results and characteristics of included studies

**Fig. 1:**
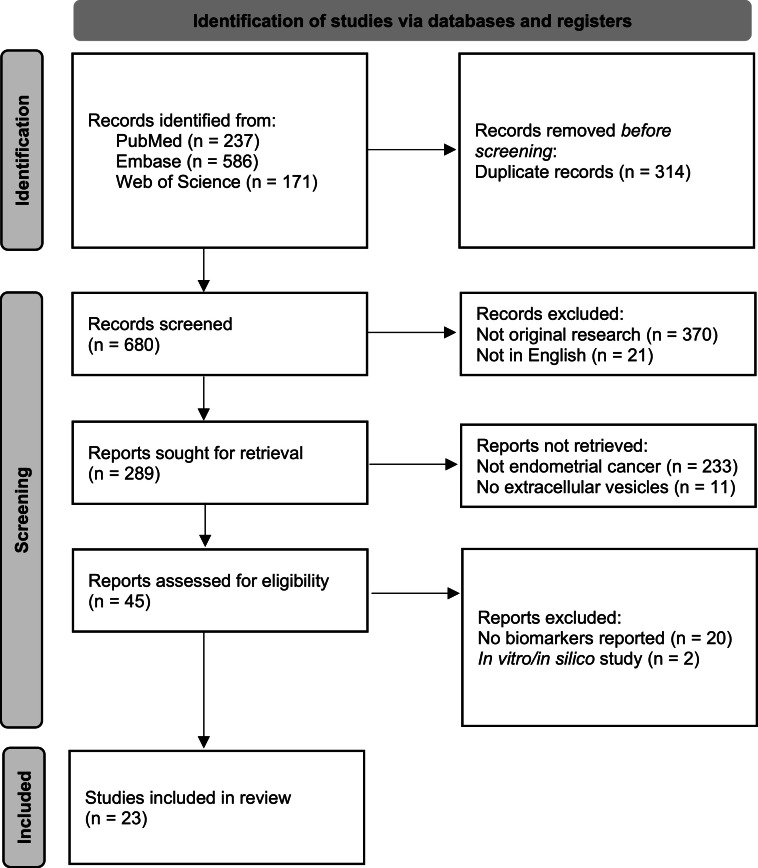
PRISMA flow diagram.

A total of 680 unique records were reviewed and 23 studies were included (Fig. 1). Details of the included studies can be seen in Supplementary File 1. Most studies were performed in either Europe (11 studies) or Asia (10 studies), while two studies were from North America. Studies included between one and 140 endometrial cancer cases and two and 118 control subjects. The description of endometrial cancer cases varied between studies. Thirteen studies provided additional clinical (e.g., age) and histopathological (e.g., subtype, grade, stage) characteristics of cases [[18], [19], [20], [21], [22], [23], [24], [25], [26], [27], [28], [29], [30]], eight studies provided limited information [[31], [32], [33], [34], [35], [36], [37], [38]] and two studies did not provide any additional clinical or histopathological information [39,40]. Control subjects also varied between studies. Controls in 21 studies were comprised of healthy females [[18], [19], [20], [21], [22], [23], [24], [25], [26],[28], [29], [30], [31], [32], [33], [34], [35], [36], [37], [38], [39]], of which seven studies specified that this included females with a benign gynecological condition [[29], [30], [31], [32],^34^,^36^,^38^]. One study which comprised a case of endometrial cancer after breast cancer included cases of non-recurrent breast cancer as controls [40]. Another study included males and females with cirrhosis as controls [27]. In addition to endometrial cancer cases and controls, five studies also included females with ovarian cancer [22,29,31,34,36].

Most studies (14 studies) used the term ‘exosomes’ to describe the particles of interest, while the remaining studies used ‘microparticles’, ‘microvesicles’ or ‘extracellular vesicles’. Since none of these studies mentioned capturing extracellular vesicles in their process of biogenesis, we have used the broad term ‘extracellular vesicles’ to describe them. Studies did also not differentiate between whether biomarkers under investigation were located inside or outside of extracellular vesicles, hence all biomarkers are considered to be associated with extracellular vesicles.

**Fig. 2:**
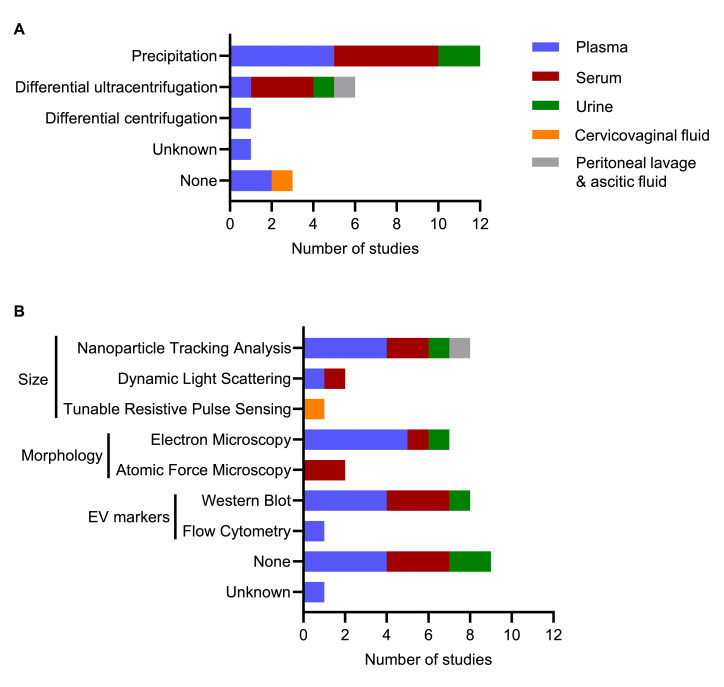
**Isolation and characterization of extracellular vesicles.** Methods for (a) isolating and (b) characterizing extracellular vesicles by biofluid.

The most common biofluid used to analyze extracellular vesicles was blood, with ten studies using plasma [18,19,21,23,24,26,32,33,36,40] and eight studies using serum [20,22,25,28,31,34,35,37]. Three studies used urine [29,30,39], one study used cervicovaginal fluid [38] and one study used peritoneal lavage from endometrial cancer cases and ascitic fluid from controls [27]. Most studies (17 studies) provided basic information about biofluid collection, processing and storage [[18], [19], [20],[22], [23], [24], [25], [26], [27], [28], [29], [30],^35^,^36^,[38], [39], [40]]. Only three studies reported time to processing [23,25,26]. Six studies did not report any pre-analytical variables [21,[31], [32], [33], [34],^37^]. No study reported fasting status. The most common method to isolate extracellular vesicles was by precipitation using commercial kits or reagents (12 studies) [18,20,22,23,25,26,[28], [29], [30],^32^,^33^,^35^], followed by differential ultracentrifugation (6 studies) [19,27,31,34,37,39] or differential centrifugation (1 study) [36] (Fig. 2a). One study did not describe how extracellular vesicles were isolated [21]. Three studies did not isolate extracellular vesicles prior to analyzing them [24,38,40]. Of the 20 studies that isolated extracellular vesicles, 13 characterized vesicles based on size (Nanoparticle Tracking Analysis, dynamic light scattering, tunable resistive pulse sensing), morphology (electron microscopy, atomic force microscopy) and/or presence of extracellular vesicle markers (CD63, CD9, CD81, TSG101 (tumor susceptibility 101), PDCD6IP (programmed cell death 6 interacting protein)) [[18], [19], [20],^23^,^27^,^28^,[31], [32], [33], [34], [35],^38^,^39^] (Fig. 2b). Only seven of these studies characterized size, morphology and protein composition of extracellular vesicles by multiple complementary techniques [18,19,23,[32], [33], [34],^39^]. Using these characterization methods, these studies identified their extracellular vesicle preparations to be enriched in small extracellular vesicles (<200 nm diameter).

Here, the key findings from all studies are reported according to biomarker type. For this systematic review, biomarkers that are associated with the presence of endometrial cancer are discussed as diagnostic biomarkers and biomarkers that are associated with prognosis or prognostic features are discussed as prognostic biomarkers. No study explored extracellular vesicles as any other type of biomarker.

### Risk of bias

**Fig. 3:**
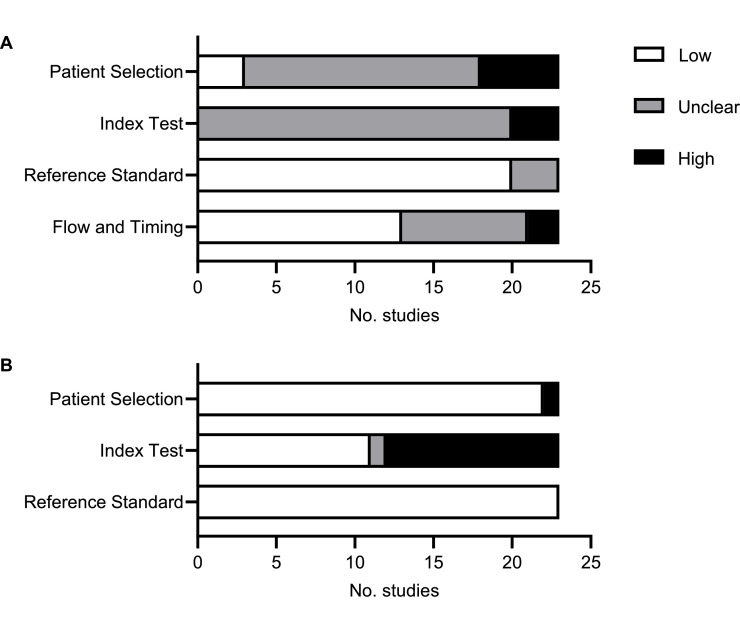
**Risk of bias assessment.** Assessment of studies for (a) risk of bias and (b) applicability concerns using the QUADAS-2 tool.

Two studies showed risk of bias in one domain, ten studies in two domains and eleven studies in more than two domains (Supplementary File 2). All studies showed either high (3 studies) or unclear risk of bias (20 studies) for the domain of index test, with no study specifying whether biomarker tests were performed blinded to diagnosis (Fig. 3a). This domain also had the highest risk for applicability concerns (Fig. 3b).

### Extracellular vesicles as diagnostic biomarkers for endometrial cancer

All 23 studies investigated extracellular vesicles as diagnostic biomarkers for endometrial cancer and 18 of these studies reported significant findings. Diagnostic biomarkers included extracellular vesicle levels and characteristics, miRNAs, proteins, circular RNAs (circRNAs) and transfer RNAs.

#### Extracellular vesicle levels and characteristics

Six studies reported that levels of extracellular vesicles were elevated in endometrial cancer cases versus controls [18,24,32,34,35,38], while two studies reported no difference [27,36]. Specifically, enumeration of particle numbers using Nanoparticle Tracking Analysis identified higher levels of extracellular vesicles in plasma [18] and serum [35]; while using tunable resistive pulse sensing, higher levels of extracellular vesicles were identified in cervicovaginal fluid [38]. Specific subpopulations of extracellular vesicles were enumerated in serum and higher levels of tissue factor-positive, CD14-positive (monocyte-derived) and CD144-positive (endothelial-derived) large extracellular vesicles were identified in endometrial cancer cases [24]. One study each reported higher levels of extracellular vesicles in females with endometrial cancer by estimation of total protein in plasma extracellular vesicles [32] or by activity of sodium-potassium adenosine triphosphatase in serum extracellular vesicles [34]. In addition to extracellular vesicles levels, Rojalin et al. (2020) reported serum extracellular vesicle surface characteristics by Surface-enhanced Raman scattering as a potential biomarker and innovative platform for endometrial cancer detection with 100 % sensitivity, 99.2 % specificity and 99.4 % accuracy, albeit in a very small sample size (three cases of endometrial cancer) [31].

#### Extracellular vesicle-associated miRNAs

The most frequently studied diagnostic biomarkers were miRNAs (9 studies). Two studies performed untargeted RNA sequencing of plasma extracellular vesicles to identify differentially abundant miRNAs in a discovery cohort; top hits were then assessed by targeted methods in a validation cohort [21,23]. However, only one of these studies had independent discovery and validation cohorts [23], whilst the other study did not report all methods or data, limiting its utility [21]. Two studies performed panel sequencing of extracellular vesicle-associated miRNAs, one from urine and the other from peritoneal lavage [27,39]. Two studies performed panel sequencing of miRNAs in either plasma or serum before validating miRNAs of interest in extracellular vesicles isolated from these biofluids [25,26]. Three studies performed quantitative reverse transcriptase PCR to analyze select miRNAs in either serum or urine extracellular vesicles [22,29,30].

Six studies reported that extracellular vesicle-associated miRNAs were differentially abundant between endometrial cancer cases and controls [[21], [22], [23],[25], [26], [27]]. Two studies reported no significant findings, however both studies were by the same authors, appear to include many of the same patients and analyzed the same miRNAs [29,30]. The significance of one study is uncertain as p-values were not reported [39]. Seventeen miRNAs were reported to be differentially abundant between endometrial cancer cases and controls in at least two independent studies (Table 1). Eight of these miRNAs were consistently differentially abundant: miR-15a-5p and miR-21–3p were elevated while miR-26a-5p, miR-130a-3p, miR-139, miR-219a-5p, miR-222–3p and miR-885 were decreased in endometrial cancer cases versus controls [21,23,27]. miR-200b-3p was reported to be decreased in endometrial cancer cases versus controls in two independent studies [23,27] but was not differentially abundant in two other non-independent studies [29,30]. Eight miRNAs, miR-20b-5p, miR-101, miR-126, miR-151a-3p, miR-182–5p, miR-194–5p, miR-451a and miR-1180–3p were reported to be differentially abundant between endometrial cancer cases and controls, however, they were elevated in one study but decreased in another study [21,23,[25], [26], [27]]. A further 36 miRNAs were reported to be elevated and 103 were decreased in endometrial cancer cases versus controls in one study each (Supplementary File 3) [[21], [22], [23],^27^].

**Table 1:** Extracellular vesicle-associated miRNAs that are differentially abundant in **endometrial cancer versus controls in multiple studies.** miRNAs are listed along with the biofluid from which extracellular vesicles were isolated and method of isolation.

miRNA	−3p	−5p	None
miR-15a		*Elevated:* Plasma (precipitation, unknown) [21,23]	
miR-20b		*Elevated:* Serum (precipitation) [25] *Decreased:* Peritoneal lavage (ultracentrifugation) [27]	
miR-21	*Elevated:* Peritoneal lavage (ultracentrifugation) & Plasma (precipitation) [23,27]	*Elevated:* Peritoneal lavage (ultracentrifugation) [27] *Decreased:* Plasma (unknown) [21] *Non-significant:* Urine (precipitation) [29,30]	
miR-26a		*Decreased:* Peritoneal lavage (ultracentrifugation) & Plasma (unknown) [21,27]	
miR-101	*Elevated:* Plasma (unknown) [21] *Decreased:* Peritoneal lavage (ultracentrifugation) [27]	*Decreased:* Plasma (precipitation) [23]	
miR-126	*Elevated:* Peritoneal lavage (ultracentrifugation) [27]	*Elevated:* Peritoneal lavage (ultracentrifugation) [27] *Decreased:* Plasma (precipitation) [23]	
miR-130a	*Decreased:* Peritoneal lavage (ultracentrifugation) & Plasma (precipitation) [23,27]		
miR-139	*Decreased:* Plasma (precipitation) [23]	*Decreased:* Peritoneal lavage (ultracentrifugation) [27]	
miR-151a-3p	*Decreased:* Peritoneal lavage (ultracentrifugation) [27]	*Elevated:* Plasma (precipitation) [26]	
miR-182		*Elevated:* Plasma (unknown) [21] *Decreased:* Peritoneal lavage (ultracentrifugation) [27]	
miR-194		*Elevated:* Plasma (precipitation) [23] *Decreased:* Peritoneal lavage (ultracentrifugation) [27]	
miR-200b	*Decreased:* Peritoneal lavage (ultracentrifugation) & Plasma (precipitation) [23,27] *Non-significant:* Urine (precipitation) [29,30]		
miR-219a		*Decreased:* Peritoneal lavage (ultracentrifugation) & Plasma (precipitation) [23,27]	
miR-222	*Decreased:* Peritoneal lavage (ultracentrifugation) & Plasma (precipitation) [23,27]		
miR-451a			*Elevated:* Plasma (precipitation) [23] *Decreased:* Peritoneal lavage (ultracentrifugation) [27]
miR-885	*Decreased:* Plasma (precipitation) [23]	*Decreased:* Peritoneal lavage (ultracentrifugation) [27]	
miR-1180	*Elevated:* Plasma (precipitation) [23] *Decreased:* Peritoneal lavage (ultracentrifugation) [27]		

Only two studies reported AUC for extracellular vesicle-associated miRNAs as diagnostic biomarkers. Zhou et al. (2021) reported AUCs of 0.61 for miR-106b-5p, 0.68 for miR-107 and 0.82 for miR-15a-5p in an independent validation cohort – this was increased to 0.87 when all three miRNAs were combined [23]. Roman-Canal et al. (2019) reported that eight miRNAs, miR-10b-5p, miR-34b-3p, miR-34c-3p, miR-34c-5p, miR-200b-3p, miR-383–5p, miR-449b-5p and miR-2110 each had an AUC between 0.90 and 0.96, sensitivity between 0.72 and 0.90, and specificity between 0.79 and 0.93 [27]. In this study, miRNA performance was reported in the same cohort in which they were discovered.

#### Extracellular vesicle-associated proteins

Eight studies explored extracellular vesicle-associated proteins as diagnostic biomarkers for endometrial cancer, of which seven studies reported significant findings. Two studies performed untargeted proteomics of either plasma or serum extracellular vesicles to identify differentially abundant proteins in a discovery cohort before validating top hits by targeted methods in a validation cohort [20,32]. However, only one of these studies had independent discovery and validation cohorts [32]. One study performed proteomic analysis of serum and then validated top hits in extracellular vesicles isolated from this biofluid [28]. One study performed proteomic analysis of extracellular vesicles purified from two endometrial cancer cell lines and then validated top hits in plasma extracellular vesicles [19]. Four studies used targeted methods (Western blot and/or ELISA) to analyze select proteins in either plasma or serum extracellular vesicles [18,33,34,40].

Twelve proteins were reported to be differentially abundant between endometrial cancer cases and controls in at least two independent studies (Table 2). Only one of these proteins, LGALS3BP (galectin 3 binding protein), was consistently reported across multiple independent studies to be elevated in endometrial cancer cases versus controls [19,20,32]. Furthermore, Song et al. (2021) reported that LGALS3BP had an AUC of 0.74 in an independent validation cohort [32]. SERPINA5 (serpin family A member 5) was discovered and subsequently validated in an independent cohort to be elevated in endometrial cancer cases versus controls in two studies by the same authors (AUC = 0.77) [32,33]. The other ten proteins, AFM (afamin), APOA1 (apolipoprotein A1), AZGP1 (alpha-2-glycoprotein 1 zinc-binding), C1QC (complement C1q C chain), C4BPB (complement component 4 binding protein beta), FGA (fibrinogen alpha chain), IGKV3–20 (immunoglobulin kappa variable 3–20), IGLV3–19 (immunoglobulin lambda variable 3–19), ORM2 (orosomucoid 2) and SERPING1 (serpin family G member 1) were reported to be differentially abundant between endometrial cancer cases and controls in more than one study, however, they were elevated in one study but decreased in another study [20,32]. Two studies investigated ANXA2 (annexin A2) as a diagnostic biomarker, but only one of these studies reported that it was elevated in endometrial cancer cases versus controls (AUC of 0.75 was reported in a validation cohort that also included all samples from the discovery cohort) [18], whilst it was not differentially abundant in the other study [19]. SERPINC1 (serpin family C member 1) was also investigated as a diagnostic biomarker in two studies, but one study reported that it was decreased in endometrial cancer cases versus controls [32], whilst it was not differentially abundant in the other study [28].

**Table 2:** **Extracellular vesicle-associated proteins that are differentially abundant in endometrial cancer versus controls in multiple studies.** Proteins are listed along with the biofluid from which extracellular vesicles were isolated and method of isolation.

Protein	Abundance
AFM	*Elevated:* Serum (precipitation) [20] *Decreased:* Plasma (precipitation) [32]
APOA1	*Elevated:* Serum (precipitation) [20] *Decreased:* Plasma (precipitation) [32]
AZGP1	*Elevated:* Serum (precipitation) [20] *Decreased:* Plasma (precipitation) [32]
C1QC	*Elevated:* Serum (precipitation) [20] *Decreased:* Plasma (precipitation) [32]
C4BPB	*Elevated:* Serum (precipitation) [20] *Decreased:* Plasma (precipitation) [32]
FGA	*Elevated:* Serum (precipitation) [20] *Decreased:* Plasma (precipitation) [32]
IGKV3–20	*Elevated:* Plasma (precipitation) [32] *Decreased:* Serum (precipitation) [20]
IGLV3–19	*Elevated:* Plasma (precipitation) [32] *Decreased:* Serum (precipitation) [20]
LGALS3BP	*Elevated:* Plasma (precipitation, ultracentrifugation) & Serum (precipitation) [19,20,32]
ORM2	*Elevated:* Serum (precipitation) [20] *Decreased:* Plasma (precipitation) [32]
SERPINA5	*Elevated:* Plasma (precipitation) [32,33]
SERPING1	*Elevated:* Serum (precipitation) [20] *Decreased:* Plasma (precipitation) [32]

In a study that comprised females with non-recurrent breast cancer as controls, plasma HSP70 (heat shock protein 70; both free and vesicular) was elevated in a female who was diagnosed with endometrial cancer after breast cancer [40]. As there was only one case of endometrial cancer in this study, it was not possible to perform statistical analyses. A further 84 extracellular vesicle-associated proteins were reported to be elevated and 47 were decreased in endometrial cancer cases versus controls in one study each (Supplementary File 4) [18,20,28,32,34]. Of these 132 proteins, five had some form of performance reported. Sommella et al. (2022) reported that in a multivariate logistic regression model, PF4V1 (platelet factor 4 variant 1), APOE (apolipoprotein E) and HBD (haemoglobin subunit delta) had a combined AUC of 0.84, sensitivity of 82.9 % and specificity of 71.4 % to identify endometrial cancer [20]. In this study, biomarker performance was reported in a validation cohort that also included all samples from the discovery cohort. Ura et al. (2021) reported that ITIH4 (inter-alpha-trypsin inhibitor heavy chain 4) and C1R (complement C1r) had a combined AUC of 0.81, sensitivity of 80.0 % and specificity of 86.7 % [28]. In this study, biomarker performance was reported in the same cohort in which they were discovered.

#### Other extracellular vesicle-associated biomarkers

One study examined circRNAs in extracellular vesicle preparations. Xu et al. (2018) performed untargeted RNA sequencing of serum extracellular vesicles and identified 275 circRNAs that were differentially abundant between endometrial cancer cases and controls [35]. circ_0109046 and circ_0002577 were both verified by quantitative reverse transcriptase PCR to be elevated in endometrial cancer cases versus controls, although this verification cohort also included all the samples from the discovery cohort. Qian et al. (2022) reported that the transfer RNA-derived small RNA tRF-20-S998LO9D was decreased in serum extracellular vesicles from endometrial cancer cases versus controls with an AUC of 0.77, although performance was reported in the same cohort in which tRF-20-S998LO9D was discovered [37].

### Extracellular vesicles as prognostic biomarkers for endometrial cancer

Seven studies investigated extracellular vesicles as prognostic biomarkers for endometrial cancer, six of which reported significant findings, though each biomarker was reported to be significant in only one study (Supplementary File 5) [18,[20], [21], [22], [23],^32^]. Prognostic biomarkers included extracellular vesicle-associated miRNAs and proteins.

#### Extracellular vesicle-associated miRNAs

Three studies investigated extracellular vesicle-associated miRNAs as prognostic biomarkers. High levels of miR-15a-5p and miR-93 and low levels of miR-26a-5p and miR-205 were associated with poor prognostic features, including larger tumors, deep infiltration and advanced stage disease, in one study each [[21], [22], [23]]. Only one study reported AUC; Wang et al. (2022) reported that plasma extracellular vesicle miR-26a-5p had an AUC of 0.83 for detecting lymph node metastases in endometrial cancer (AUC was reported in a validation cohort that also included all samples from the discovery cohort) [21]. One study reported molecular classifiers; Zhou et al. (2021) demonstrated that high levels of plasma extracellular vesicle miR-15a-5p were associated with p53-abnormal disease [23], which is associated with poor prognosis. Zheng et al. (2020) reported that high levels of serum extracellular vesicle miR-93 (in the discovery cohort, AUC was 0.99, sensitivity 93 % and specificity 97 %) or low levels of miR-205 were associated with reduced overall survival [22]. The authors also reported that miR-93 was elevated and miR-205 was decreased in smokers versus non-smokers.

#### Extracellular vesicle-associated proteins

Four studies investigated extracellular vesicle-associated proteins as prognostic biomarkers. Sommella et al. (2022) reported that high levels of serum extracellular vesicle CA1 (carbonic anhydrase 1), HBD and PF4V1 were associated with early-stage disease (combined AUC = 0.98, sensitivity = 100.0 %, specificity = 86.1 %) and APOE was associated with advanced stage disease (AUC = 0.81, sensitivity = 83.3 %, specificity = 68.6 %) – performance was reported in a validation cohort that also included all samples from the discovery cohort [20]. Herrero et al. (2019) reported that plasma extracellular vesicle ANXA2 was elevated in endometrial cancers with poor prognostic features, including non-endometrioid, high-grade, advanced stage disease with a high risk of recurrence [18]. Song et al. (2021) reported that plasma extracellular vesicle ACTG1 (actin gamma 1), CFHR1 (complement factor H related 1), IGHV1–2 (immunoglobulin heavy variable 1–2), IGHV3–43 (immunoglobulin heavy variable 3–43), IGKV3–20, LGALS3BP, LRRC28 (leucine rich repeat containing 28), ORM1 (orosomucoid 1), PPBP (pro-platelet basic protein) and PRDX6 (peroxiredoxin 6) were elevated and CFD (complement factor D), DCD (dermcidin), PGLYRP2 (peptidoglycan recognition protein 2), PKD1L2 (polycystin 1 like 2) and POLK (DNA polymerase kappa) were decreased in endometrial cancer cases with metastatic disease [32]. Mariscal et al. (2019) explored the association between plasma extracellular vesicle levels of LGALS3BP and survival but found none [19].

## Discussion

This study systematically reviewed the evidence for extracellular vesicles as biomarkers for endometrial cancer. We assessed all biomarker types, providing a comprehensive overview of the current state of extracellular vesicle biomarker research in endometrial cancer. We identified 23 studies, 16 of which investigated extracellular vesicles as diagnostic biomarkers and seven studies investigated extracellular vesicles as both diagnostic and prognostic biomarkers. No study investigated extracellular vesicles as susceptibility/risk, monitoring, predictive, response or safety biomarkers.

Ten biomarkers were consistently reported as being differentially abundant between endometrial cancer cases and controls in multiple independent studies and hence may be putative diagnostic biomarkers: levels of extracellular vesicles [18,24,32,34,35,38], LGALS3BP [19,32], miR-15a-5p [21,23] and miR-21–3p [23,27] were elevated, while miR-26a-5p [21,27], miR-130a-3p [23,27], miR-139 [23,27], miR-219a-5p [23,27], miR-222–3p [23,27] and miR-885 [23,27] were decreased in endometrial cancer cases versus controls. Weighting was not applied to biomarker selection as all included studies displayed risk of bias in at least one domain. No biomarker was reported as prognostic in more than one study, most likely reflecting the paucity of research in this area.

Levels of extracellular vesicles have been reported to be elevated in both females and males with cancer, including breast and pancreatic cancer, compared to healthy controls [6,41]. LGALS3BP has been reported to be one of the most abundant proteins in extracellular vesicles isolated from individuals with cancer [5]. LGALS3BP is an extracellular matrix protein that can be recovered with extracellular vesicles as per Minimal Information for Studies of Extracellular Vesicles (MISEV2023 and previous versions) recommendations and hence is a general protein marker for extracellular vesicles [9]. It therefore appears that levels of extracellular vesicles and LGALS3BP are general markers of cancer and may not be suitable as specific biomarkers for endometrial cancer.

Consistent with the findings that the expression of miR-21–3p is elevated while miR-26a-5p, miR-130a-3p, miR-139 and miR-219a-5p are decreased in extracellular vesicle preparations from endometrial cancer cases versus controls, all five miRNAs are reported to be similarly regulated in endometrial cancer tissue compared to normal tissue [[42], [43], [44], [45]]. This indicates that the expression of these miRNAs in extracellular vesicle preparations might reflect tissue expression and hence these five extracellular vesicle-associated miRNAs could be biomarkers for endometrial cancer. However, as none of the studies differentiated whether miRNAs are encapsulated within extracellular vesicles or are co-isolates, it cannot be discounted that they are contaminants of the extraction process and hence may not be clinically useful biomarkers for endometrial cancer. By contrast, miR-15a-5p is elevated in extracellular vesicle preparations from endometrial cancer cases versus controls but is reported to be decreased in endometrial cancer tissue compared to normal tissue [42,46]. Similarly, miR-222–3p is decreased in extracellular vesicle preparations from endometrial cancer cases versus controls but is reported to be elevated in endometrial cancer tissue compared to normal tissue [47]. miR-885 is decreased in extracellular vesicle preparations from endometrial cancer cases versus controls, however, it is reported to be elevated in serous but not endometrioid endometrial cancer compared to benign endometrium [48]. These inconsistencies raise questions about the validity of extracellular vesicle-associated miR-15a-5p, miR-222–3p or miR-885 as biomarkers for endometrial cancer. They also suggest that cancer cells may package miRNAs and other molecules into extracellular vesicles which do not necessarily represent the abundance of molecules in the cell of origin [49].

There are numerous limitations of the studies included in this systematic review. Successful detection and development of extracellular vesicle biomarkers depend on various factors such as type of biological material, pre-analytical variables (e.g., procedure for sample collection and handling prior to extracellular vesicle isolation), method used for extracellular vesicle isolation and biomarker detection, as well as sample size and population. Variation in these areas is anticipated to contribute to a lack of overlap in biomarkers between studies. Most of the included studies provided basic information about biofluid collection, processing and storage, but in the absence of reporting of all pre-analytical variables, including fasting status, biofluid transport conditions and length of storage, no study was fully compliant with international consensus recommendations on extracellular vesicle research (MISEV2023 and previous versions) [9]. Many of the included studies also lacked additional clinical and histopathological information about endometrial cancer cases and had limited information about controls. Extracellular vesicle number and composition have been reported to be impacted by pre-analytical variables as well as other factors such as population demographics (e.g., age, race, sex) and lifestyle (e.g., physical activity, smoking) [[50], [51], [52]]. These need to be considered to ensure extracellular vesicle biomarkers are related to endometrial cancer and not due to these confounding factors.

Most studies included in this systematic review enriched extracellular vesicles from biofluids, thereby increasing the ratio of extracellular vesicles to non-vesicular extracellular particles. Twelve studies employed polymer-based precipitation techniques available as a commercial kit in which extracellular particles are aggregated before being pelleted at low-speed centrifugation. Precipitation based methods provide a less intensive method of extracellular vesicle separation which can be easily scalable for commercial applications [53]. They also provide higher yield or recovery of extracellular vesicles from the starting material; however, they do not exclusively isolate extracellular vesicles. Free proteins, lipoproteins and other extracellular macromolecular complexes that aggregate non-specifically with extracellular vesicles are also co-isolated during the precipitation process, resulting in lower extracellular vesicle purity or particle to protein ratio [54]. Six studies used differential ultracentrifugation to isolate extracellular vesicles in which larger or denser extracellular vesicles are pelleted at 10,000 g to 20,000 g and smaller or lighter extracellular vesicles at 100,000 g to 200,000 g. However, pellets from ultracentrifugation are contaminated with particles of a similar sedimentation coefficient as extracellular vesicles and hence pure populations of extracellular vesicles fail to separate [53]. This is particularly relevant when working with biofluids that are enriched in non-vesicular extracellular particles such as blood which contains free soluble proteins including albumin, immunoglobulins, fibrinogen and lipoproteins [5,9,55]. These proteins overlap in size and density with extracellular vesicles and outnumber extracellular vesicles by orders of magnitude, making them major contaminants and frequent confounding factors in obtaining pure extracellular vesicle preparations [56]. Co-isolated contaminants can compromise downstream analyses making detection of less abundant proteins difficult, contributing to false-positive signals and impacting quantification of extracellular vesicles, making it difficult to attribute detected biomolecules exclusively to extracellular vesicles [57]. For example, abundant free soluble proteins can interfere with mass spectrometry-based detection and reduce signal specificity for extracellular vesicle-derived content. Additionally, extended ultracentrifugation steps can lead to vesicle deformation or rupture, potentially altering their cargo and function. Therefore, while these methods are efficient for extracellular vesicle enrichment, they do not provide a pure extracellular vesicle population and should be interpreted with caution when used for biomarker discovery.

To understand the association between extracellular vesicles and potential biomarkers of disease, MISEV recommends that appropriate negative controls such as non-vesicular extracellular particles or whole biofluids also be evaluated [9]. Only three of the studies included in this systematic review assessed biomarkers of interest in both whole biofluids and extracellular vesicles isolated from these biofluids and none analyzed non-vesicular extracellular particles. One study initially identified six miRNAs in whole serum that were differentially abundant between endometrial cancer cases and controls (miR-20b-5p, miR-143–3p, miR-195–5p, miR-204–5p, miR-423–3p and miR-484) which, when re-assessed in extracellular vesicles, only miR-20b-5p was differentially abundant [25]. Similar results were also reported in whole plasma and urine where only one or two of the originally identified miRNAs or proteins were also differentially abundant in extracellular vesicles [26,28]. This emphasizes that some biomarkers may be free-circulating or present in non-vesicular extracellular particles and co-isolated with extracellular vesicles as artifacts of the extraction process rather than genuinely encapsulated within extracellular vesicles. To attribute a biomarker function to extracellular vesicles instead of co-isolates, pure preparations of extracellular vesicles with proper characterization are required. Only seven of the included studies characterized extracellular vesicles by multiple complementary techniques in accordance with MISEV recommendations [9]. Nonetheless, enrichment and characterization alone do not confirm extracellular vesicle purity and co-isolation of non-extracellular vesicle material remains a significant concern. Biomarkers identified from such preparations must be interpreted cautiously as they may originate from co-purified contaminants. However in some cases, particularly when diagnostic accuracy is the priority, biomarker utility may not require strict confinement within extracellular vesicles. If sufficient sensitivity and specificity can be demonstrated, complete extracellular vesicle purification may not be necessary [9].

Other limitations of studies included in this systematic review are that studies by the same authors appear to not be independent of each other but rather reported different or even the same biomarkers from the same patients [25,26,29,30,32,33]. Four studies performed untargeted analyses (two studies each performed untargeted RNA sequencing [21,23] or proteomics [20,32]), however, only two of these studies had independent discovery and validation cohorts [23,32]. The development and clinical application of diagnostic and prognostic biomarkers require rigorous validation in independent cohorts to ensure their reliability and utility. We also identified limited information related to the performance of the biomarkers discussed in this systematic review (summarized in Supplementary File 6). Sensitivity and specificity are fundamental metrics that determine the ability of biomarkers to correctly identify the presence (true positives) and absence (true negatives) of the disease. Without robust validation of these key characteristics, it is challenging to assess the effectiveness and accuracy of biomarkers in a clinical setting.

In summary, current studies investigating extracellular vesicles as minimally invasive biomarkers for endometrial cancer are limited by numerous factors impacting the quality and reliability of results. These include small sample sizes with inadequate information about cases and controls; under-reporting of technical variables relating to biofluid collection and processing that can affect extracellular vesicle quantity and quality; limited use of appropriate methodologies to isolate and characterize extracellular vesicles and identify contaminants that can impact on extracellular vesicle yield and biomarker identification; and insufficient assessment of biomarker performance metrics in independent cohorts. ISEV has released minimal information recommendations (MISEV2023 and previous versions) and research groups have released recommendations on extracellular vesicle research in endometrial cancer to account for and reduce variability between studies [9,58]. Compliance with the rigor and standardization framework put forward by ISEV, combined with complete and transparent reporting of case and control populations and methods, will enhance reproducibility in extracellular vesicle biomarker identification and validation. Future studies should focus on comparative evaluations of extracellular vesicle isolation methods to determine how different protocols affect biomarker profiles and signal attribution. Determining whether biomarkers are inside extracellular vesicles, membrane-bound or merely co-isolated is essential for functional interpretation. Mechanistic studies exploring the selective packaging of biomolecules into extracellular vesicles and their physiological relevance will provide further insight into their diagnostic utility. The development and broad adoption of standardized protocols aligned with MISEV recommendations will enhance reproducibility and facilitate the clinical translation of extracellular vesicle-based biomarkers [9].

There are also some limitations of this systematic review. The absence of full biomarker lists provided by some studies means that there may be more extracellular vesicle-associated biomarkers than are discussed here. Also, the heterogeneity of included studies, many of which had small sample sizes, and the lack of reporting of effect sizes, standard errors or confidence intervals prevented us from performing a meta-analysis.

Despite the limitations, the advantages of extracellular vesicles as biomarkers for endometrial cancer are potentially clinically changing. Liquid biopsies are minimally invasive and are more suitable to be taken at multiple timepoints for disease monitoring than a tissue biopsy. Furthermore, biofluids are homogenous, avoiding issues such as tumor heterogeneity. Hoshino et al. (2020) demonstrated that specific combinations of extracellular vesicle proteins can distinguish between pancreatic, lung, breast and colorectal cancers and mesothelioma, indicating that it is possible to derive cancer-specific signatures [5]. In the future, extracellular vesicles may be used to screen females for endometrial cancer, triage symptomatic females for endometrial biopsy, improve risk stratification to assist in clinical decision making, monitor disease progression and ultimately replace invasive endometrial tissue collection procedures.

## Conclusions

This systematic review identified ten extracellular vesicle-associated biomarkers that were consistently reported in multiple independent studies to be differentially abundant between endometrial cancer cases and controls. Levels of extracellular vesicles, LGALS3BP, miR-15a-5p and miR-21–3p were elevated and miR-26a-5p, miR-130a-3p, miR-139, miR-219a-5p, miR-222–3p and miR-885 were decreased in endometrial cancer cases versus controls. Of these biomarkers, miR-21–3p, miR-26a-5p, miR-130a-3p, miR-139 and miR-219a-5p may be the most promising diagnostic biomarkers as their expression in extracellular vesicle preparations appears to reflect that in endometrial tissue. No biomarker was reported as prognostic in more than one study. However, study quality and limited adherence to MISEV recommendations are of significant concern. Furthermore, as none of the included studies differentiated whether biomarkers of interest were located within extracellular vesicles as opposed to artifacts of the extraction process, all biomarkers can only be considered to be associated with extracellular vesicles. Large, high-quality studies in well documented populations that use consistent biofluids and methods to isolate, characterize and analyze extracellular vesicles and adhere to MISEV recommendations are needed to demonstrate the clinical potential of extracellular vesicles as minimally invasive biomarkers to improve diagnosis and treatment planning for females with endometrial cancer.

## Data availability

All data generated during this study are included in this article and its supplementary files.

## Funding statement

EB is supported by the Cherish Women’s Cancer Foundation. SN is supported by a June Summers Research Fellowship. AO and CS are supported by the National Health and Medical Research Council (APP1194334, APP1195451). This work is supported by the National Health and Medical Research Council (APP2014633). The funders had no role in the design of the study; collection, analysis or interpretation of data; writing of the report or decision to submit the article for publication.

## CRediT authorship contribution statement

**Eva Baxter:** Writing – original draft, Visualization, Methodology, Formal analysis, Data curation, Conceptualization. **Soumyalekshmi Nair:** Writing – original draft, Visualization, Formal analysis. **Zoe West:** Writing – review & editing. **Carlos Salomon:** Writing – review & editing, Supervision, Funding acquisition. **Andreas Obermair:** Writing – review & editing, Supervision, Funding acquisition.

## Declaration of competing interest

The authors declare that they have no known competing financial interests or personal relationships that could have appeared to influence the work reported in this paper.

## Appendix Supplementary materials

Image, application 1

Image, application 2

Image, application 3

Image, application 4

Image, application 5

Supplementary material Image, application 6
